# Digital mandala coloring as a public mental health tool: psychophysiological benefits and the mediating role of flow in anxiety reduction among university students

**DOI:** 10.3389/fpubh.2026.1743520

**Published:** 2026-04-08

**Authors:** Xiangyu Li, Yinan Li, Xuan Zhang, Haimin Zhang, Wenyu Wu

**Affiliations:** 1 School of Art, Southeast University, Nanjing, China; 2 School of Design, Anhui Polytechnic University, Wuhu, China; 3 School of Mechanical Engineering, Southeast University, Nanjing, China

**Keywords:** anxiety, art therapy, digital mandala coloring, flow, physiological measurement, psychological health

## Abstract

**Background:**

Anxiety is becoming more common among young people globally. At the same time, digital mental health services are developing rapidly. Digital mandala coloring applications have become popular tools for managing anxiety. However, it remains unclear if they are effective as psychological tools, and their mechanism of action lacks scientific verification. This study aims to investigate the effectiveness of digital mandala coloring in relieving anxiety and the role of flow in its mechanism.

**Methods:**

This study involved 60 university students across two experiments employing coloring intervention using physiological and psychological measurements. Experiment 1 compared the differences in anxiety relief and level of flow state between digital and traditional paper-based mandala coloring. Experiment 2 further explored the impact of two mainstream interaction modes, sliding coloring and gamified coloring, in digital mandala coloring on user experience and therapeutic effect. All participants underwent anxiety induction before a 30-min coloring activity. State anxiety and physiological indicators were measured at baseline (T1), after anxiety induction (T2), and after the coloring intervention (T3). Flow state level was measured at T3.

**Results:**

Digital mandala coloring demonstrated efficacy in reducing self-reported state anxiety, with no significant difference to the traditional method. However, it induced a higher level of flow state and demonstrated stronger physiological calming effects, specifically greater reductions in heart rate and skin conductance level. Experiment 2 indicated that, compared to the gamified coloring condition, sliding digital mandala coloring led to greater anxiety reduction, a higher level of flow state, and more pronounced improvements in heart rate and skin conductance level recovery. Crucially, the flow state was found to fully mediate the relationship between the digital coloring mode and state anxiety level. These results suggest that the anxiety-reducing effect of digital mandala coloring can be enhanced by increasing immersion and fostering flow.

**Conclusion:**

Digital mandala coloring is an effective anxiety intervention for university students. Its therapeutic effect can be significantly enhanced by interaction designs that improve operational fluency, as such designs promote a deeper flow state. This study provides a key insight for developing digital mental health applications: optimizing interactive fluency to promote immersive flow experience is more important than pursuing entertainment for enhancing emotional healing value. This finding points the way for applying and developing digital interventions in public health.

## 1 Introduction

The rising prevalence of anxiety among younger populations is a global issue. This phenomenon correlates with increasing rates of adolescent mental disorders. Carl Jung empirically established the anxiety-reducing effect of paper-based mandala coloring. Subsequent research confirms its superior efficacy. Empirical evidence (1–7) demonstrates that mandala coloring outperforms other coloring activities and non-artistic interventions in alleviating anxiety. Given this strong empirical support, Khademi et al. (8) conducted a clinical trial. Their study involved hospitalized COVID-19 patients. Participants engaged in 30-min mandala coloring sessions daily for six consecutive days. This intervention led to a significant reduction in their anxiety.

The COVID-19 pandemic has profoundly altered lifestyles and accelerated the global reliance on mobile devices. This shift has prompted the mental health community to embrace a new digital healthcare paradigm. Within this context, digital coloring applications have emerged as digital alternatives to paper-based mandala coloring, undergoing significant development in recent years. Digital mandala coloring offers distinct advantages, including greater accessibility, immediate engagement, and diverse interactive features. These benefits contribute to its growing preference over traditional methods. Market surveys indicate the availability of over one hundred digital mandala coloring apps across major platforms. Among these, the popular application “Unicorn” has recorded more than 430,000 downloads. This substantial user base demonstrates the considerable market presence and promising growth potential of digital mandala coloring. Although these applications are now widely marketed as “art therapy” tools, their actual psychological therapeutic effects and underlying mechanisms of action remain scientifically unverified.

### 1.1 Digital mandala coloring: from digital medium to therapeutic mechanism

1. How digitization influence emotional regulation processes in coloring

Early studies suggested that digital media could not satisfy clients’ sensory needs compared to traditional mediums. Some researchers even questioned whether digitally created art held therapeutic value for clients (9). However, this conclusion may no longer align with today’s globally digitalized reality. The impact of COVID-19, technological advancements, and the high digital literacy of younger generations of art therapists have gradually integrated digital tools into art therapy practices. There is now a generally open and optimistic attitude toward digital art creation (10). Sion et al. (11) found that creating art on a tablet provides stress-reducing effects similar to using oil pastels. Digital medium offers users complete flexibility in execution, thereby enhancing their sense of control and flow during the creative process (12). The latest trends indicate that users increasingly prefer various digital drawing applications when selecting mediums for artistic activities.

In studying people’s reactions in response to different art mediums, Snir and Regev’s (13) findings emphasize the importance of acknowledging how different art mediums elicit different responses in art therapy. The factors of different sensations from the art medium emphasizing different parts of the art-making experience (14). This relates to the “affordances” of medium in the digital age, which represent predictable potential for action based on a medium’s technical capabilities. It influences and constrains both actions and experiences (15). Some of the inherent properties of digital coloring could lead to novel situations in art therapy, further mediating emotional regulation processes. Firstly, the infinite reversibility in digital coloring activity is helpful to build a safe exploratory environment. The undo function in digital software was designed to correct errors quickly without disrupting workflow, that reduces users’ error-related anxiety and promotes cognitive flexibility (16). Choe (17) mentioned users with anxiety issues may benefit more from digital art therapy activities than traditional ones, with digital media may be familiar and less threatening. In addition, dynamic and instant feedback is key mechanism that enhance positive reinforcement and facilitate flow states to counteract anxiety (18). The multiple feedback in digital coloring applications is helpful for users to gain a sense of self-efficacy and empowerment, to counteract the loss of pleasure brought by anxiety (19–21). However, some physiological data still indicate that traditional art materials are more effective at activating the human brain. Paper provides a richer emotional experience, which can contribute to cognitive and emotional development of users (22). These findings indicated the importance of art materials in therapy tasks.

Digital art therapy possesses distinct advantages, but research on specific digital therapies remains limited, and only a few were focused on developing specific digital art therapy interventions or compared digital and traditional mediums (23). Indeed, there is a gap in the literature investigating the different therapeutic effects each art medium brings, especially between traditional and digital methods (13, 24). There is insufficient data concerning the therapeutic potential of digital art making and its positive outcomes. The physiological and psychological effects of digital mandala coloring therapy on users remain a question that warrants further research.

1. The development of digital mandala coloring in psychotherapy

The existing literature presents divergent conclusions regarding the emotional therapeutic effects of digital coloring. Mantzios et al. (25) suggest that digital mandala coloring provides anxiety reduction comparable to traditional paper-based methods. In contrast, Han et al. (22) found that drawing on paper activates brain regions associated with cognition and emotion more strongly than using a tablet. Additionally, Roh et al. (14) demonstrated that older adults with lower digital literacy experienced greater relief from death anxiety through traditional coloring. A major limitation of lots of existing conclusion lies in the heavy reliance on subjective self-report measures. Currently, there is a lack of objective experimental data beyond questionnaire-based studies. Most existing research depends primarily on self-reported measurements, with insufficient support from psychophysiological indicators. Furthermore, participants’ varying familiarity with digital media and the inadequate range of metrics used to compare anxiety reduction between traditional and digital coloring complicate the interpretation of results.

Despite these limitations, practical applications are advancing. For instance, South Korea has begun integrating mandala art therapy into digital platforms, offering “digital mandala” services within public mental health applications (26). It is therefore necessary to investigate the impact and mechanisms of different operations and designs within digital coloring activities on users’ emotional states. This research will enable future improvements to the operational processes of digital coloring applications, thereby enhancing both the user experience and emotional feedback.

### 1.2 Factors and mechanisms influencing user experience and psychological state in digital mandala coloring

1. The mediating role of flow experience

*Flow theory* (27) posits that tasks providing immediate feedback can enhance one’s sense of personal control, thereby facilitating flow state that help alleviate anxiety and release stress. However, existing research has generally viewed traditional coloring as a mindfulness activity that promotes mindful thinking, few of researchers focus on flow as a key characteristic to adjust users’ emotion in mandala coloring. This may be because paper-based coloring publications were initially often referred to as “mindfulness coloring books,” making research on whether mindfulness is a potential psychological change triggered by mandala coloring relatively more common.

Mindfulness is a conscious state of being attentive and aware of the present moment, while upholding a non-judgemental attitude, which is usually practiced through meditation (28–30). Mantzios and Giannou (31) found that participants engaged in digital mandala coloring demonstrated significantly higher level of state mindfulness compared to those using paper-based methods. However, his study did not establish mindfulness as the key mechanism behind anxiety reduction in digital mandala coloring. Another study found that while mandala coloring can enhance mindfulness and reduce anxiety, it did not perform significant advantages than other ordinary activities, and mindfulness has not been proven to play a mediating or moderating role in this context (3). Compared to mindfulness, which involves perceiving and being aware of present-moment pleasure and activity, flow is a state of total immersion. The high interactivity and appeal of mandala coloring activities help participants detach their thoughts from anxiety through distraction mechanisms (32). The diversion of users’ attention from anxiety to coloring activity serves as the foundation for extending into a state of flow. Therefore, perhaps flow is the crucial factor in mandala coloring.

Some research have already verified whether flow is a key factor in mandala coloring activities, but limited to traditional paper-based mode. Mihaly (33) suggested that coloring facilitates dialogue with the subconscious through meditation, enabling entry into a “flow” state that reduces anxiety and tension. Forkosh (34) similarly demonstrated that paper-based mandala coloring significantly enhanced participants’ flow state level. Cai Xuyan (35) further found that while mandala coloring elevated both flow and mindfulness, only flow mediated the relationship between paper-based coloring and anxiety reduction. Therefore, it can be inferred that digital mandala coloring may function through a mechanism similar to its traditional counterpart, by enhancing flow states to ultimately alleviate anxiety.

1. The impact mechanism of interactive operations

Research on how different coloring operations and interface designs in digital mandala coloring affect user experience and psychological states remains scarce, primarily due to insufficient data on its positive effects. In studies on digital mobile media user experience, Huang (36) found that higher level of mobile interactivity facilitate greater flow experience. Since operational fluency reflects the ease of mental simulation during interaction (37), higher fluency strengthens the connection between psychological activity and actual behavior (38). Thus, fluency can enhance interactivity with mobile devices, leading to stronger immersion and flow experience (39). Conversely, gamified designs and dynamic feedback, which characterized as “task-switching” elements, may increase visual fixation pressure and distract attention (40), thereby reducing immersion. It can be inferred that operational fluency in digital mandala coloring influences users’ sense of immersion, thereby altering flow level and subsequently affecting anxiety reduction.

Compared to the singular mode of traditional coloring, complex operational and gamified designs in current digital mandala apps may hinder anxiety relief and emotional relaxation. Therefore, it is reasonable to hypothesize that simple sliding coloring operations provide a more relaxing and pleasant experience than gamified interactions, primarily by evoking higher flow state through streamlined operation.

### 1.3 Application of physiological indicators in psychotherapy

Research has confirmed that anxiety can induce elevated heart rate (HR) (41, 42). Turturro and Drake (43) experimentally demonstrated that coloring effectively reduces anxiety and lowers participants’ HR. Heart rate variability (HRV) also serves as an indicator of emotional state, with higher HRV associated with improved emotion regulation (44) and considered a marker of psychological health (45, 46). Key HRV metrics include time-domain indices (e.g., rMSSD, SDNN, pNN50) and frequency-domain indices (e.g., HF, LF). rMSSD has been widely adopted by researchers to measure HRV. Specifically, rMSSD is used to evaluate parasympathetic nervous system (PNS) activity, which represents relaxation and recovery (47). Higher rMSSD values indicate stress reduction, making them suitable for reflecting the immediate emotional impact of the intervention activities in this study. Significant decreases in rMSSD is closely linked to anxiety disorders (48). Among HRV time-domain analysis metrics, HF serves as an indicator of parasympathetic nervous system (PNS) activity (49), LF serves as an indicator of sympathetic nervous system (SNS) activity. LF has been validated as an effective indicator of flow states, lower LF indicated a higher level of flow state, demonstrating the provision of more attentional resources (50). Therefore, both rMSSD and LF indices were incorporated into the experimental measurement scope. The inclusion of HRV indices facilitates comparative analysis in the statistical assessment of cardiac flow state level.

Skin conductance level (SCL) is another reliable measure of psychological stress, with higher SCL observed under high-stress conditions (51). SCL data are widely used in art therapy research to evaluate whether specific art forms or activities alleviate anxiety (52–54). In typical stress-induction experiments, participants first undergo an anxiety-provoking task, followed by an art-based intervention during a recovery period. This design creates alternating high-stress and low-stress phases, during which participants’ SCL should correspondingly fluctuate between high and low levels (54). Therefore, we selected skin conductance level (SCL), heart rate (HR), and specific HRV indices (rMSSD and LF) as key physiological measures. These indicators will provide robust physiological evidence to compare the psychophysiological effects of mandala coloring in anxiety reduction.

### 1.4 Current study and research hypotheses

Building on existing research, this study focuses on university students, a demographic highly familiar with digital media. Firstly, it investigates the psychophysiological effects of digital versus traditional mandala coloring in anxiety reduction, and examines the changes of flow state. Then, by comparing two digital coloring methods (sliding coloring V.S gamified coloring), this research aims to elucidate their relationships with anxiety alleviation and flow state. The findings will enrich embodied cognition theory regarding the relationship between operational fluency and flow state, while providing practical insights for optimizing user experience and emotional outcomes in digital mandala applications.

This study extends previous work in several key aspects. First, we compare the psychophysiological effects of digital and traditional mandala coloring using multiple indicators: skin conductance level (SCL), heart rate (HR), and heart rate variability (HRV). Since generalized anxiety disorder includes symptoms of muscular tension (43), and anxiety triggers both psychological and physiological responses, a comprehensive assessment of both dimensions is essential to determine whether digital mandala coloring can effectively regulate anxiety.

Second, to address gaps in existing digital mandala research, our second experiment treats different digital coloring operations as independent variables. This allows us to identify factors influencing users’ flow state, thereby informing future design improvements in digital mandala applications. Based on a review of relevant literature, we propose the following hypotheses:

*H1: Digital mandala coloring reduces anxiety among university students.*

*H2: Different digital coloring operations differentially affect anxiety reduction, with sliding coloring producing more significant anxiety relief than gamified coloring.*

*H3: In digital mandala coloring, flow state mediates the anxiety reduction effect.*

To test these hypotheses, Experiment 1 will compare the anxiety reduction effects of digital versus traditional mandala coloring. Experiment 2 will further compare how two digital operation types (sliding versus gamified coloring) relate to anxiety alleviation and level of flow state.

**Figure 1:**
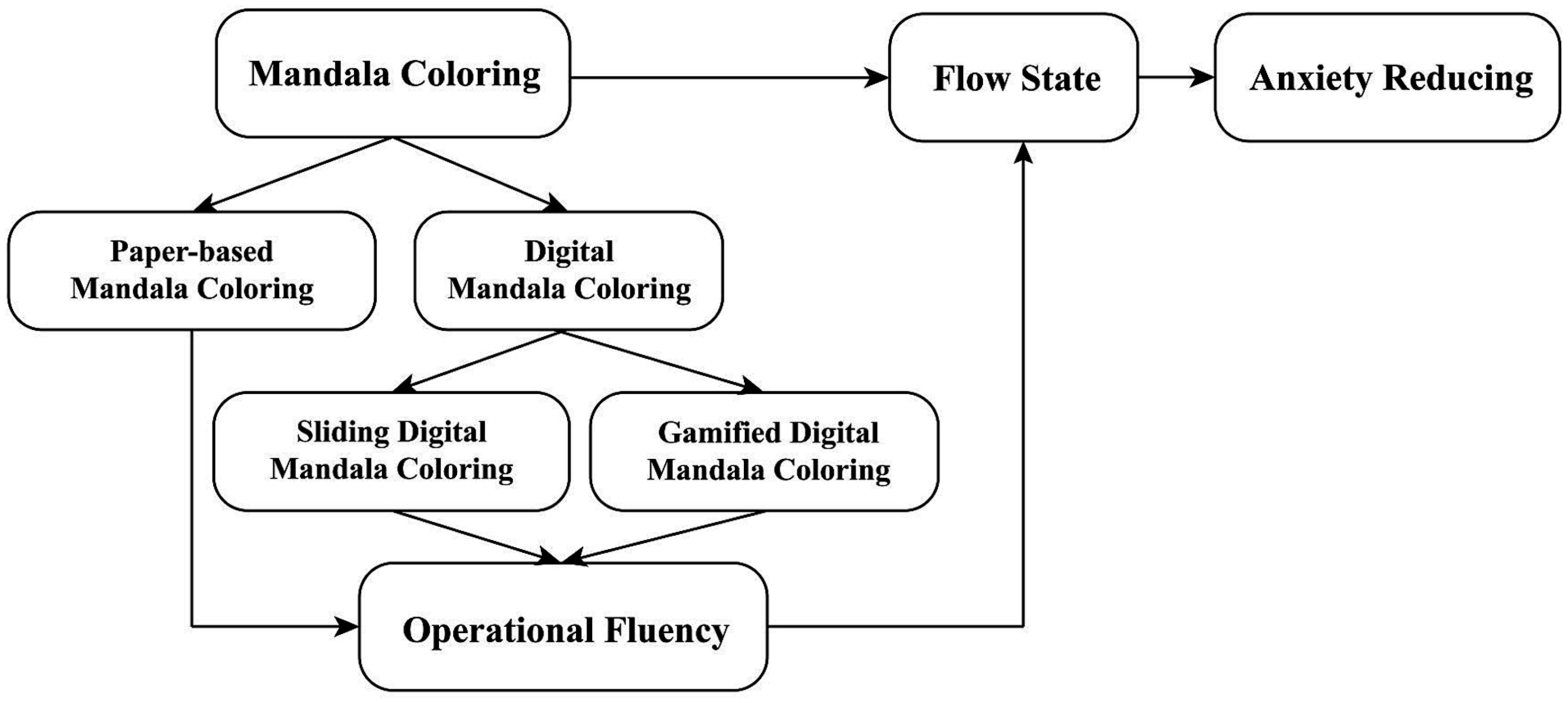
Hypothetical model: the hypothesis is different types of mandala coloring influence operational fluency, which determines the level of flow state, ultimately effect anxiety reducing.

Since coloring shows limited effects on temporary anxiety in resting participants (55, 56), and significant anxiety reduction from mandala coloring occurs mainly post-induction (57), anxiety will be measured at three time points: baseline (T1), post anxiety induction (T2), and post coloring activity (T3). The theoretical model of this study is shown in Figure 1.

## 2 Materials and methods

### 2.1 Tools

The State Anxiety Inventory (SAI) was used to assess state anxiety, defined as a subjective and transient experience of tension. State anxiety is a temporary emotional condition characterized by physiological arousal and conscious feelings of apprehension, worry, and nervousness (58). In this study, the SAI was administered at three time points (T1, T2, T3). Each item is rated on a 4-point scale from 1 (not at all) to 4 (almost always), with positively worded items reverse-scored. Higher total scores indicate higher levels of anxiety. The scale demonstrates good reliability (Cronbach’s *α* = 0.91).

The Flow State Scale (FSS) (59) was used to evaluate participants’ flow state level during the coloring activity. This 36-item scale requires participants to recall their experience during mandala coloring and rate the frequency and intensity of each statement (e.g., “I was not concerned with what others may have been thinking of me”) on a 5-point scale from 1 (never) to 5 (always). The total score reflects the overall flow state level, with higher scores indicating greater flow state. The scale has an internal consistency coefficient of 0.88. It was administered only at T3.

Psychophysiological Measures. Heart Rate (HR), Heart Rate Variability (HRV) and Skin Conductance Level (SCL) were recorded using the BIOPAC MP150-BioNomadix wireless psychophysiological recording system. Electrodes placed at different body surface locations were used to measure HRV, SCL, and HR. HR was measured in beats per minute based on the average of interbeat intervals. HRV was measured with the utilization of electrodes and a respiration strap connected to an encoder. These measures were recorded into a computer and analyzed by AcqKnowledge 4.2 software. SCL was measured using disposable electrodes on the index and middle fingers of the non-dominant hand, hooked up to a polygraph. The skin conductance channel was analyzed as the average level (SCL, in μS).

These measurements except flow state were taken for participants in all three conditions at the beginning of the experiment for 2 min (Time 1), after anxiety induction for 2 min (Time 2), and after coloring for 2 min (Time 3).

### 2.2 Coloring materials

#### 2.2.1 Experiment 1

Participants were randomly assigned to two groups, either a group with traditional paper-based mandala coloring condition or a digital coloring condition using a mobile application. The digital group used the professional drawing application “Procreate” on 12-inch iPad Pro with Apple Pencil. This application provides basic sliding coloring function using Apple Pencil that closely simulates traditional paper-based coloring. Participants were instructed to use only the Apple Pencil for sliding coloring and were prohibited from using other painting tools such as the paint bucket. Participants had the option to change the line size, zoom in and out with two fingers, erase, undo and create different colors with a digital color wheel. For the traditional coloring materials, participants received a printed A4 (210mm × 297 mm or 8.27 × 11.69 inches) sheet with a standardized black-outline mandala template. Everyone could choose from various coloring materials, including oil pastels, markers, and colored pencils (minimum 36 colors available). The same mandala template was used across both conditions, either pre-loaded into the Procreate application or printed on A4 paper. All participants colored the identical mandala pattern for the same duration to ensure experimental consistency.

#### 2.2.2 Experiment 2

The same blank mandala pattern from Experiment 1 was used. Participants were randomly assigned to one of two groups with different digital coloring applications on mobile devices. Sliding coloring group used the professional drawing application “Procreate” on 12-inch iPad Pros with Apple Pencil, as described in Experiment 1. Gamified coloring group used a coloring application that provides gamified interaction besides basic sliding coloring function. In addition to simple slide operations, the application incorporated gamified elements including bomb tools and paint bucket functions. These features required participants to engage in increased tap and click operations alongside basic slide coloring. The two interactive coloring modes were designed to operationalize distinct user experience paradigms. To control variables, Experiment 2 continued using the sliding mandala coloring application and operational procedures employed in Experiment 1. The “Sliding” mode emphasized continuous manual control for filling regions, simulating a traditional coloring experience. In contrast, the “Gamified” mode incorporated elements typical of game design. Specifically, based on traditional sliding operation, it added discrete point-and-click filling with structured goals and performance feedback. The key operational differences between two groups are summarized in Table 1.

**Table 1:** Operation of “gamified coloring group” versus “sliding coloring group”.

Group	Sliding coloring group	Gamified coloring group
Interaction mode	Discrete selection and click: Beyond standard sliding coloring, users can employ tools like the “Paint Bucket” in application to instantly fill enclosed areas with preselected colors via clicking, or the “Bomb” tool enables random filling of enclosed regions.	Continuous sliding: Users wield the Apple Pencil to slide the tip across the screen, directly applying color to blank mandala patterns. This method needs manual control over fill areas and speed.
Interaction feedback	Explicit, task-oriented: Provide immediate feedback (e.g., animated cues) upon filling each area. Display a completion progress bar. Coloring activities accumulate in-game experience points and level-up points.	Implicit, process-oriented: Feedback primarily stems from tactile and visual sensations triggered by the coloring action itself. No external progress indicators.
Task mode	Clear, quantifiable sub-goals: Tasks are broken down into a series of progressively completed sections.	Open-ended, process-focused: Tasks are structured as continuous, free-form coloring activities without segmentation.
Progress indicator	Explicit: A visual progress bar or percentage counter displays overall completion.	Implicit: Progress is conveyed solely through changes in the overall pattern’s state.

### 2.3 Participants

Participants were 60 university students (36 females and 24 males) ranging in age from 18 to 30 years (M = 25.67; [SD] = 3.87) who were recruited and received research credit as part of a course requirement. All participants were currently enrolled in bachelor’s or higher degree programs, reported no physical or psychological disorders, and showed no evidence of significant mental health conditions or social functioning impairments.

Regarding artistic background, 33.33% of participants had some art experience (defined as at least 1 year of systematic art appreciation or formal training in any art form), while the majority (66.67%) reported no formal art education. The study protocol was approved by the Institutional Review Board of the authors’ institution, and all participants provided written informed consent before participation.

### 2.4 Procedure

#### 2.4.1 Randomization

In each experiment, participants were randomly assigned to either group A or group B using a random sampling method. Each group consists of 15 randomly selected participants. They were randomly assigned to one of the two experimental conditions, assignment was performed using a computer-generated block randomization sequence with a fixed block size of 4 by an experimenter. In experiment 1, group A is paper-based mandala coloring group, and group B is digital mandala coloring group. In experiment 2, group A is sliding digital mandala coloring group, and group B is gamified digital mandala coloring group.

#### 2.4.2 Experiment procedure

The session was a single continuous experimental session lasting approximately 40 min, including baseline assessment (T1), anxiety induction, pre-test assessment (T2), mandala coloring intervention, and post-test assessment (T3).

All experimental sessions were conducted individually in a private, quiet room. The procedure began with a 2-min baseline period during which participants remained seated quietly while baseline psychophysiological measurements were recorded (T1). Participants then completed the State Anxiety Inventory (SAI) for the first time (T1). During the anxiety induction phase, participants were instructed to recall and write about the most anxiety-provoking event they had ever experienced. Following this writing task, participants completed the SAI while psychophysiological measurements were recorded for the second time (T2).

Inducing anxiety of subjects through writing task is commonly employed in mandala coloring experiments. This method was introduced by Curry and Kasser (5), demonstrated that a 4-min writing exercise could induce changes in mood. This method has been consistently applied in subsequent mandala coloring studies (7, 60). Consequently, this study adopted the writing expression approach during the anxiety induction phase. After the T1 inventory, to induce an anxious emotional state in the study participants, they were asked to recall a prior event in their lives that they felt most fearful and then to write down their experiences on a piece of unlined A4-sized paper (21.0 cm × 29.7 cm). Experimenter read verbatim instructions to participants: “Please recall a recent personal event that caused you high anxiety or stress, and write about its occurrence, your feelings, and thoughts in as much detail as possible.”Then left 4 min to participants finish their writing tasks. The whole induction period lasted for approximately 5 min. Immediately after the 5 min, the participants completed the SAI for the second time (T2).

**Figure 2:**
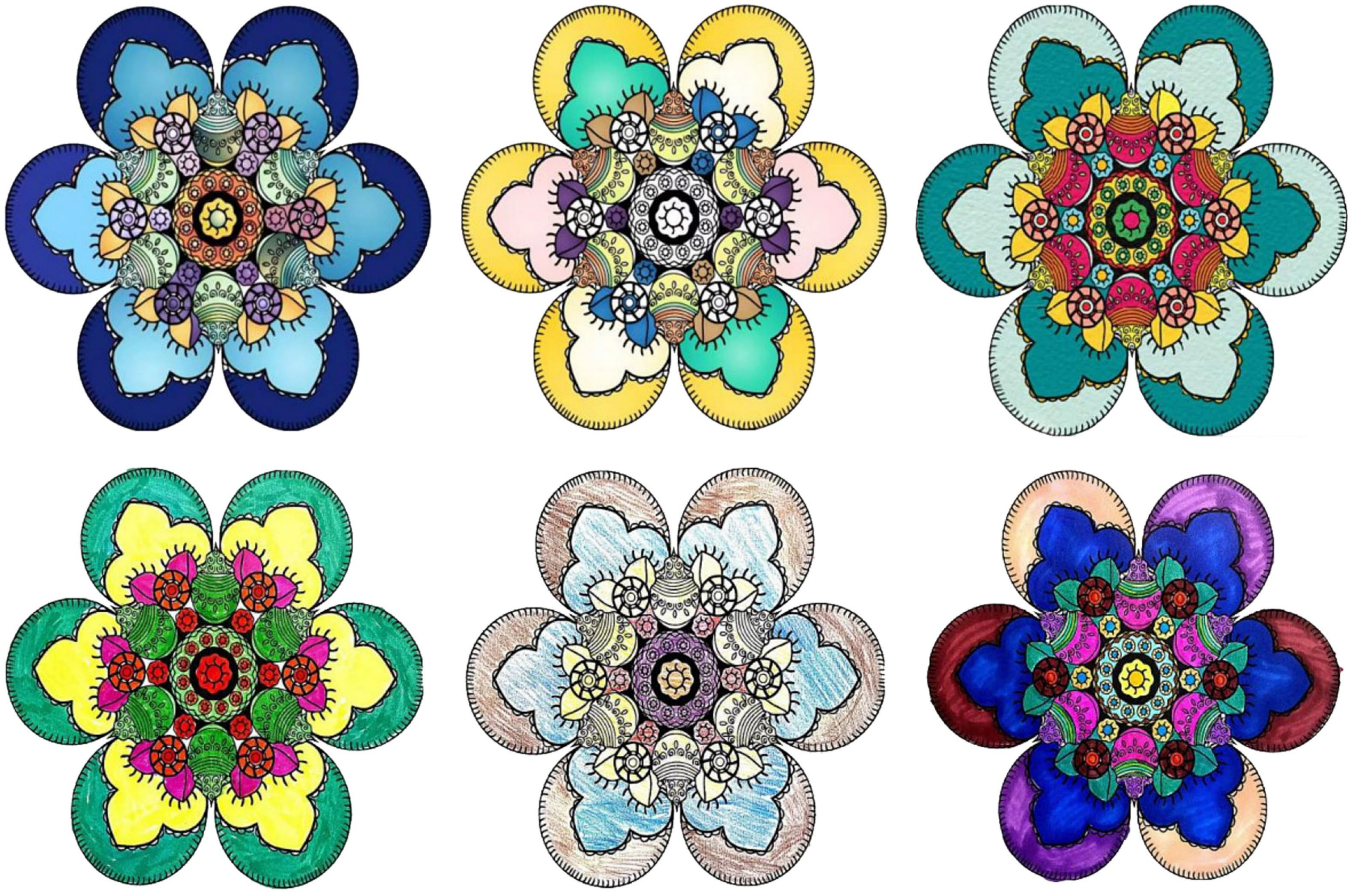
Examples of participants’ coloring results.

After anxiety induction, each participant engaged in a 30-min coloring activity according to their assigned condition. Psychophysiological measurements were recorded after coloring activities finished (T3). Participants filled out the SAI for the third time (T3) and the Flow State Scale. Figure 2 below shows some of the pictures colored by participants in each experiment.

### 2.5 Statistical analysis

Data analysis was performed using SPSS 21.0 (IBM Corp., Armonk, NY, USA). Descriptive statistics, difference tests, and repeated-measures analysis of variance (ANOVA) were conducted. Sphericity tests were applied with corrections: when Mauchly’s W > 0.75, the Huynh-Feldt correction was used; when *W* < 0.75, the Greenhouse–Geisser correction was applied. For the HRV performance during the tasks, we found that some of the HRV parameters (LF) and SCL did not satisfy the normalized distribution from the results of the Shapiro–Wilk test (*p* < 0.05). Due to the skewed distribution of SCL and HRV recordings, parameters of SCL and LF data were log-transformed by taking the natural logarithm (Ln). Therefore, we calculated the Ln SCL and Ln LF parameters to satisfy the normalized distribution, then submitted them to all the parameters into two-way within-subjects repeated-measures of analysis of variance (ANOVA).

**Figure 3:**
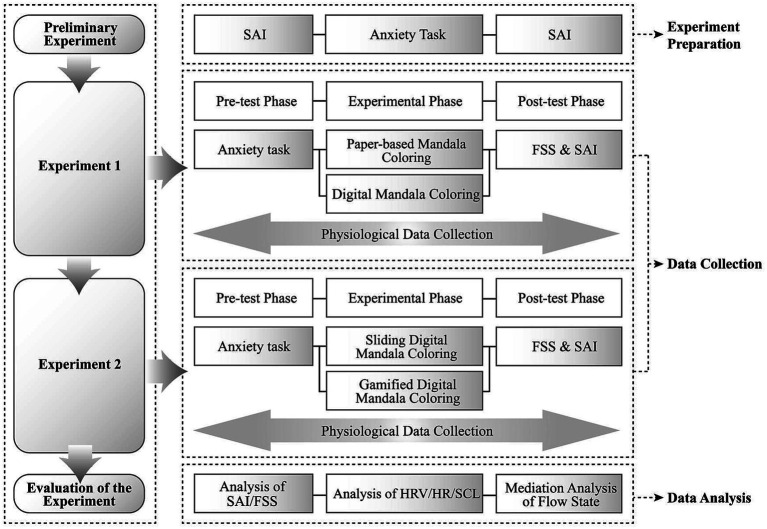
Structure flowchart.

The overall framework of the experiment is shown in Figure 3.

## 3 Results

### 3.1 Data analysis for Experiment 1

Before conducting more analysis, it’s necessary to perform homogeneity tests on each measurement for both groups at baseline (T1) using the independent samples *t-*test method to ensure the comparability of the two datasets. All homogeneity test results are presented in Table 2. The statistical results confirm that all data collected at the same time point are homogeneous, allowing subsequent statistical analyses to proceed.

**Table 2:** Descriptive statistics for each dependent variable by group and measurement time (M ± SD).

Var.	*t*-test (T1)		Paper group (*N* = 15)			Digital group (*N* = 15)		
	*t*	*p*	T1	T2	T3	T1	T2	T3
SA	−0.228	0.821	32.67 ± 9.76	44.00 ± 10.77	31.6 ± 7.11	33.33 ± 5.70	46.27 ± 9.72	27.67 ± 5.42
SCL	−0.285	0.778	1.88 ± 0.60	2.56 ± 0.40	2.12 ± 0.71	1.94 ± 0.64	2.83 ± 0.37	2.22 ± 0.62
HR	−0.227	0.822	80.80 ± 17.80	86.53 ± 17.68	82.13 ± 16.22	82.00 ± 10.17	87.87 ± 11.12	79.00 ± 9.92
rMSSD	1.726	0.095	39.60 ± 10.49	25.20 ± 7.78	37.20 ± 11.84	35.80 ± 12.84	27.27 ± 7.72	38.20 ± 9.91
LF	−0.267	0.792	6.12 ± 0.69	6.86 ± 0.50	6.61 ± 0.48	5.95 ± 1.33	6.27 ± 0.91	6.47 ± 0.96

**Figure 4:**
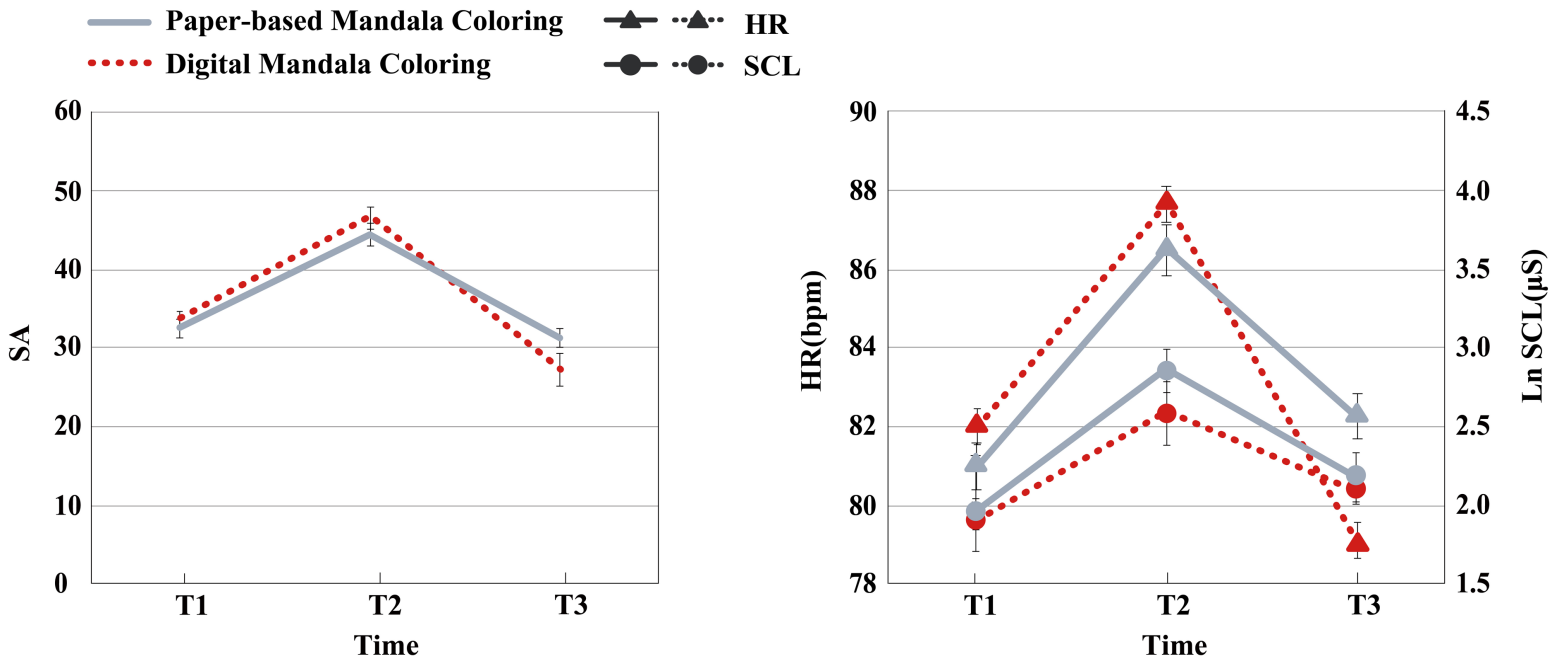
Means on the state anxiety, Ln skin conductance level, and heart rate in experiment 1 at Time 1, Time 2, and Time 3 by condition. Error bars represent standard errors. The results indicated that both paper-based mandala coloring and digital mandala coloring are effectively in alleviating anxiety, with both activities reducing HR and SCL. Participants’ HR after digital mandala coloring were significantly lower than baseline.

Table 2 and Figure 4 presents the means and SDs and SEs of state anxiety (SA) and physiological measures at T1, T2, and T3 by condition.

#### 3.1.1 Effect on condition of decreasing anxiety and increasing flow state

Repeated measures ANOVA (Table 3) was employed with group and measurement time as independent variables and state anxiety as the dependent variable. Repeated measures ANOVA showed that anxiety increased from T1 to T2 (*t*(56) = −9.05, *p <* 0.001, 95%CI [−17.556, −9.844]), and anxiety decreased from T2 to T3 (*t*(56) = 8.44, *p* < 0.001, 95%CI[−22.000, −11.800]). The effect of time was significant [*F*(2,56) = 70.373, *p* < 0.001, η_p_^2^ = 0.915]. The interaction between group and measurement time was not significant [*F*(2,56) = 3.95, *p* = 0.051, η_p_^2^ = 0.04]. An independent sample *t-*test was conducted on the difference of baseline and after anxiety induction scores (pre-post score) for state anxiety between the paper-based and digital groups. Results indicated no significant difference in pre-post score changes between the two groups [*t*(28) = 1.601, *p =* 0.121]. This demonstrated that anxiety induction effectively elevated anxiety levels, while activity intervention effectively alleviated anxiety. No significant difference was found between the two activities in their efficacy for alleviating anxiety.

**Table 3:** Repeated measures analysis of variance (ANOVA) for SA, SCL and HR (Experiment 1).

Var.	SA			SCL			HR		
	*F*	*p*	η_p_^2^	*F*	*p*	η_p_^2^	*F*	*p*	η_p_^2^
Group	0.026	0.875	0.002	0.740	0.397	0.021	0.002	0.965	0.000
Measurement time	70.373	0.000***	0.915	30.830	0.000^***^	0.524	22.327	0.000^***^	0.775
Group * Measurement time	2.315	0.138	0.263	3.324	0.043^*^	0.106	7.469	0.007^**^	0.535

*p < 0.05, **p < 0.01, ***p < 0.001.

An independent sample *t-*test was conducted on the flow state scores of the paper-based group and the digital group. Results indicated that the mean score and standard deviation (SD) for the paper-based group was 49.667 ± 8.910, while the digital group’s mean score and SD was 55.667 ± 5.327. The difference in flow state scores between the two groups was statistically significant [*t*(28) = −2.239, *p =* 0.033 < 0.05], with the digital group demonstrating significantly higher flow state scores than the paper-based group.

#### 3.1.2 Physiological benefits of different coloring mode

1. Effect of condition on decreasing skin conductance level

Results of paired sample *t* test indicated that, compared to T1, SCL significantly increased in both groups at T2 and decreased significantly from T2 to T3 [*t*(14) = −7.186, *p* < 0.001, *d* = 1.855; t(14) = −6.387, *p* < 0.001, *d* = 1.645]. Repeated measures ANOVA (Table 3) was conducted on SCL across time points for both groups, with SCL as the dependent variable and group and time as independent variables. Results indicated a significant main effect of time [*F*(2, 56) = 30.830, *p* < 0.001, η_p_^2^ = 0.524]. The main effect of group was not significant [*F*(1, 28) = 0.740, *p =* 0.397, η_p_^2^ = 0.021]. The interaction between group and time was significant [*F*(2, 56) = 3.324, *p =* 0.043 < 0.05, η_p_^2^ = 0.106]. An independent sample *t* test was conducted on the difference in SCL (T3-T2) between the paper-based and digital groups before and after the coloring activity. Results indicated significant differences in SCL between groups [*t*(28) = 2.431, *p =* 0.022 < 0.05; *t*(28) = 2.605, *p =* 0.015 < 0.05], with the paper-based group exhibiting significantly higher mean SCL than the digital group. This indicated that the digital mandala coloring group demonstrated a more pronounced effect in reducing participants’ SCL.

1. Effect of condition on decreasing heart rate

Paired samples *t-*test results indicated that, compared to T1, HR significantly increased in both groups at T2, and decreased significantly from T2 to T3 [*t*(14) = −5.467, *p* < 0.001, *d* = 1.049; *t*(14) = −4.231, *p =* 0.001 < 0.01, *d* = 1.387]. Repeated measures analysis of variance (ANOVA) (Table 3) was conducted on HR across measurement time points for both groups, with HR as the dependent variable and group and measurement time as independent variables. Results indicated a significant main effect of measurement time [*F*(2,56) = 22.327, *p* < 0.001, η_p_^2^ = 0.775]; The main effect of group was not significant [*F*(1,28) = 0.002, *p =* 0.965, η_p_^2^ < 0.001]; the interaction effect between group and measurement time was significant [*F*(2,56) = 7.469, *p =* 0.007 < 0.01, η_p_^2^ = 0.535]. Further simple effect analysis revealed: participants in the paper-based mandala coloring group showed no significant difference in HR between T1 and T3 (*p =* 0.474), while participants in the digital mandala coloring group had significantly lower HR at T3 compared to T1 (*p =* 0.040 < 0.05). This indicated that the digital mandala coloring group demonstrated a more pronounced effect in reducing participants’ HR.

1. Effect of condition on decreasing heart rate variability

Results of paired samples *t-*test showed that there are no significant differences between the two groups at three timeline (T1, T2 and T3) with the measures of rMSSD and LF (all *p* > 0.05). With rMSSD as the dependent variable, and group and measurement time as independent variables. Results indicated a significant main effect of time [*F*(2,56) = 35.065, *p* < 0.01, η_p_^2^ = 0.556] and a significant main effect of group [*F*(1,28) = 6.920, *p =* 0.020 < 0.05, η_p_^2^ = 0.331]. The interaction between group and measurement time was not significant [*F*(2,56) = 1.070, *p =* 0.371]. With LF as the dependent variable and group and measurement time as independent variables. Results indicated a significant main effect of time [*F*(2,56) = 8.068, *p* < 0.01, η_p_^2^ = 0.224], while the main effects of group and the interaction between group and measurement time were not significant (*p =* 0.941, *p =* 0.151).

### 3.2 Conclusion of Experiment 1

In summary, the results of Experiment 1 confirmed Hypothesis 1. The results of Experiment 1 indicated that both paper-based mandala coloring and digital mandala coloring are effectively in alleviating anxiety [*t*(28) = 1.601, *p =* 0.121], with both activities reducing HR and SCL. However, no significant difference was observed in participants’ heart rate before and after paper-based mandala coloring (*p =* 0.474), while participants’ HR after digital mandala coloring were significantly lower than baseline (*p =* 0.040 < 0.05). They also exhibited higher level of flow state than paper-based group [Paper group: M ± SD = 49.667 ± 8.910, digital group: M ± SD = 55.667 ± 5.327, *t*(28) = −2.239, *p =* 0.033 < 0.05].

The effect of anxiety reduction on participants after digital mandala coloring in Experiment 1 also corroborated by their flow state level, which established a research foundation for further investigating whether flow state directly correlates with digital mandala coloring and anxiety levels. Consequently, Experiment 2 would further exploring whether the anxiety-reducing and physiological alleviating effects of digital mandala coloring among university students due to its induction of heightened flow states.

### 3.3 Data analysis for Experiment 2

As mentioned before, Experiment 2 was developed to examine Hypothesis 2 and 3. We inferred that different digital mandala coloring operations differently affect the effect of therapy, with sliding coloring producing more significant anxiety relief than gamified coloring. In addition, in digital mandala coloring, flow state mediates the anxiety reduction effect.

**Figure 5:**
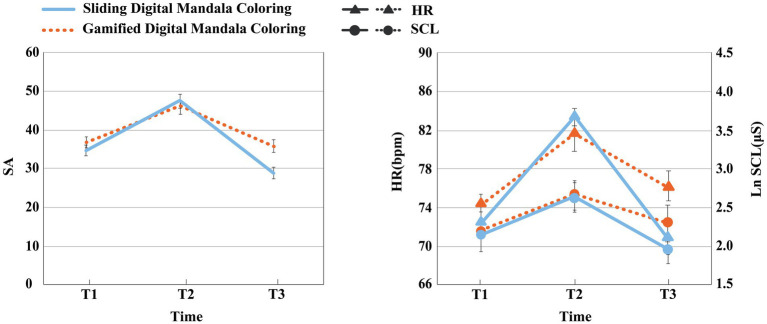
Means on the state anxiety, Ln skin conductance level, and heart rate in Experiment 2 at Time 1, Time 2, and Time 3 by condition. Error bars represent standard errors. The results indicated that sliding group demonstrated significantly greater anxiety reduction than the gamified group. Regarding physiological indicators, the sliding group also exhibited a significantly greater reduction in HR compared to the gamified group.

All homogeneity test results are presented in Table 4. The statistical results confirmed that all data collected at the same time point are homogeneous, allowing subsequent statistical analyses to proceed. Table 4 and Figure 5 presents the means and SDs and SEs of state anxiety (SA) and physiological measures at T1, T2, and T3 by condition.

**Table 4:** Descriptive statistics for each dependent variable by group and measurement time (M ± SD).

Var.	*t*-test (T1)		Sliding group (*N* = 15)			Gamified group (*N* = 15)		
	*t*	*p*	T1	T2	T3	T1	T2	T3
SA	−0.574	0.517	34.60 ± 8.67	47.47 ± 4.56	28.73 ± 5.61	36.47 ± 9.14	46.33 ± 9.19	34.80 ± 8.69
SCL	−0.490	0.628	2.18 ± 0.50	2.63 ± 0.38	1.96 ± 0.62	2.22 ± 0.62	2.69 ± 0.52	2.33 ± 0.68
HR	−0.535	0.597	72.33 ± 9.82	83.20 ± 10.62	70.60 ± 8.15	74.20 ± 9.29	81.73 ± 14.28	76.33 ± 11.59
rMSSD	0.440	0.663	40.40 ± 9.84	30.00 ± 4.19	44.20 ± 4.09	37.2 ± 6.46	35.4 ± 3.82	45.33 ± 4.58
LF	0.124	0.902	5.99 ± 0.95	5.44 ± 0.96	6.13 ± 0.89	5.82 ± 1.21	5.73 ± 1.11	6.00 ± 0.94

#### 3.3.1 Effect on condition of decreasing anxiety and increasing flow state

Repeated measures ANOVA (Table 5) was conducted with group and measurement time as independent variables and state anxiety as the dependent variable. ANOVA showed that anxiety increased from T1 to T2 (*t*(56) = −5.685, *p* < 0.001, 95%CI[−14.620, −1.394]), and decreased from T2 to T3 (*t*(56) = −4.401, *p* < 0.001, 95%CI[−11.930, −8.820]). The main effect of time was significant [*F*(2,28) = 36.619, *p* < 0.001, η_p_^2^ = 0.849]; the main effect of group and interaction between group and time was not significant. An independent sample *t* test was conducted on the pre-post change (T3-T2) difference in state anxiety between the paper-based and digital groups. Results indicated a significant difference in pre-post change scores between the two groups [*t*(28) = −2.187, *p =* 0.037 < 0.05]. This demonstrated that sliding digital mandala coloring group demonstrated significantly greater reduction in state anxiety compared to the gamified coloring group.

**Table 5:** Repeated measures analysis of variance (ANOVA) for SA, SCL and HR (Experiment 2).

Var.	SA			SCL			HR		
	*F*	*p*	η_p_^2^	*F*	*p*	η_p_^2^	*F*	*p*	η_p_^2^
Group	1.645	0.220	0.105	0.740	0.397	0.026	0.268	0.613	0.019
Measurement time	36.619	0.000^***^	0.849	30.830	0.000^***^	0.524	20.050	0.000^***^	0.755
Group * Measurement time	2.881	0.092	0.307	3.324	0.043^*^	0.106	6.019	0.014*	0.481

*p < 0.05, **p < 0.01, ***p < 0.001.

An independent sample *t-*test on flow state scores between the sliding and gamified groups revealed that the mean score and SD of sliding group participants is 55.667 ± 5.327, while the gamified group averaged 48.733 ± 9.308. The difference in flow state scores between groups was significant [*t*(28) = 2.504, *p =* 0.018 < 0.05], with the sliding group demonstrating markedly higher flow state scores than the gamified group.

#### 3.3.2 Physiological benefits of different coloring mode

1. Effect of condition on decreasing skin conductance level

Paired sample *t* test results indicated that, SCL significantly increased in both groups from T1 to T2 and decreased significantly from T2 to T3 [*t*(14) = −5.279, *p* < 0.001, *d* = 0.918; t(14) = −5.989, *p* < 0.001, *d* = 0.952]. Repeated measures analysis of variance (ANOVA) (Table 5) was conducted for both groups with SCL as the dependent variable and group and time as independent variables. Results indicated a significant main effect of time [*F*(2,56) = 30.830, *p* < 0.001, η_p_^2^ = 0.524]; The main effect of group was not significant [*F*(1, 28) = 0.61, *p =* 0.441, η_p_^2^ = 0.021]; the interaction between group and measurement time was significant [*F*(2,56) = 3.324, *p* < 0.05, η_p_^2^ = 0.106]. Further simple effects analysis revealed that both groups exhibited a common pattern of significantly higher T2 SCL than T1 and T3 (*p* < 0.005). An independent sample *t-*test was conducted on the difference in SCL between the sliding and gamified groups before and after the coloring activity (T3–T2). Results indicated significant differences in SCL between groups [*t*(28) = 5.21, *p* < 0.001, *d* = 0.89]. The decline value for the sliding group (*M* = −12.3, SD = 2.1) was significantly greater than that for the gamified group (*M* = −6.8, SD = 1.9). This indicated that the sliding digital mandala coloring group demonstrated a more pronounced effect in decreasing participants’ SCL.

1. Effect of condition on decreasing heart rate

Paired samples *t* test results indicated that, HR significantly increased in both groups from T1 to T2 [sliding group: *t*(14) = −5.026, *p* < 0.001, *d* = 2.162; gamified group: *t*(14) = −3.439, *p =* 0.004 < 0.01, *d* = 2.191], and decreased significantly from T2 to T3 [sliding group: *t*(14) = 6.562, *p* < 0.001; gamified group: *t*(14) = 3.553, *p =* 0.002 < 0.05]. An independent sample *t-*test was conducted on the pre-post change scores for state anxiety between the sliding and gamified groups. Results indicated a significant difference in pre-post changes between groups [*t*(28) = −2.688, *p =* 0.012 < 0.05], demonstrating that participants in the sliding group exhibited a significantly greater reduction in HR following the activity compared to the gamified group.

Repeated measures ANOVA (Table 5) was conducted on HR across time for both groups, with HR as the dependent variable and group and time as independent variables. Results indicated a significant main effect of measurement time [*F*(2,56) = 20.050, *p* < 0.001, η_p_^2^ = 0.755]; the main effect of group was not significant [*F*(1, 28) = 0.740, *p =* 0.397]; the interaction effect between group and measurement time was significant [*F*(2,56) = 6.019, *p =* 0.014 < 0.05, η_p_^2^ = 0.481]. Further simple effects analysis revealed: the decline in HR between T2 and T3 was significantly greater in the sliding group than in the gamified group (mean decrease: 0.670 vs. 0.363), resulting in a lower mean HR at T3 for the sliding group (1.955 ± 0.624) compared to the gamified group (2.331 ± 0.685), showing a marginally significant difference [*t*(28) = 1.829, *p =* 0.075].

Overall, participants in the sliding coloring group exhibited a greater reduction in HR than those in the gamified mandala coloring group. This indicated that the mandala coloring group demonstrated a more pronounced effect in decreasing participants’ HR level. The results showed that the flow state level in the sliding group was significantly higher than that in the gamified group, confirming that participants’ subjective reports and physiological indicators mutually corroborate each other in terms of flow state level and HR.

1. Effect of condition on decreasing heart rate variability

Results of paired samples *t* test showed that there are no significant differences between the two groups at three timeline(T1, T2 and T3) with the measures of rMSSD and LF(all *p* > 0.05). With rMSSD as the dependent variable and group and time as independent variables. Results indicated a significant main effect of time [*F*(2,28) = 7.746, *p =* 0.006 < 0.01, η_p_^2^ = 0.544]; while the interaction between group and measurement time was not significant [*F*(2,28) = 2.459, *p =* 0.124]. With LF as the dependent variable and group and time as independent variables. Results indicated a significant main effect of measurement time [*F*(2,28) = 10.304, *p =* 0.002 < 0.01, η_p_^2^ = 0.613]; the interaction between group and measurement time was not significant [*F*(2,28) = 1.970, *p =* 0.179].

### 3.4 Conclusion of Experiment 2

Overall, the results of Experiment 2 confirmed Hypothesis 2. The sliding group demonstrated significantly greater anxiety reduction and higher flow state level than the gamified group. Regarding physiological indicators, the sliding group also exhibited a significantly greater reduction in HR compared to the gamified group. Following the experimental protocol, Hypothesis 3 was further examined: whether flow mediated the effect of digital mandala coloring on anxiety, thereby verifying flow as a potential underlying mechanism for its anxiety reducing effects.

### 3.5 Mediation analysis: the role of flow state

Correlation analysis was conducted between T3 flow state level, T3 state anxiety, and group assignment (0 = Sliding digital mandala coloring group, 1 = gamified digital mandala coloring group). The correlations among variables are presented in Table 6. Both T3 state anxiety and T3 flow state level exhibited significant negative correlations with state anxiety and positive correlations with group assignment. Flow state level on T3 showed a significant negative correlation with group assignment.

**Table 6:** Correlation analysis among variables.

Variable	T3 state anxiety levels	T3 flow levels	Group
T3 state anxiety level	1		
T3 flow level	−0.685^***^	1	
Group	0.394^*^	−0.428^**^	1

*p < 0.05, **p < 0.01, ***p < 0.001.

In the mediation analysis, the independent variable was group assignment (0 = Sliding digital mandala coloring group, 1 = gamified digital mandala coloring group), treated as a dummy variable. The dependent variable was T3 state anxiety level, with T3 flow state level serving as the mediating variable. All variables except the dummy variable were standardized prior to analysis. Mediation effect results are presented in Table 7.

**Table 7:** Testing the mediating effect of flow state between groups and state anxiety.

Outcome variable	Predictor variable	*R* ^2^	*F*	*β*	*SE*	*t*	95%CI
T3 flow state level	Group	0.183	6.269^*^	−0.841	0.336	−2.504^*^	[−1.529,–0.153]
T3 state anxiety level	Group	0.482	12.561^***^	0.244	0.301	0.809	[−0.375,0.862]
	Flow state level			−0.632	0.153	−4.125^***^	[−0.947,–0.318]

*p < 0.05, **p < 0.01, ***p < 0.001.

Results revealed that group assignment was significantly associated with T3 flow state level (*β* = −0.841, *p* < 0.05). Conversely, T3 flow state level (*β* = −0.632, *p* < 0.001) was significantly associated with T3 state anxiety, whilst group did not show a significantly direct association with T3 state anxiety (*β* = 0.244, *p* > 0.05). The Bootstrap 95% confidence intervals did not include 0. The mediating effect of T3 flow state level was significant, with a mediating effect value of 0.532, 95% CI [0.756, 1.250], *p* < 0.001. The mediating effect accounted for 68.65% of the total effect.

**Figure 6:**
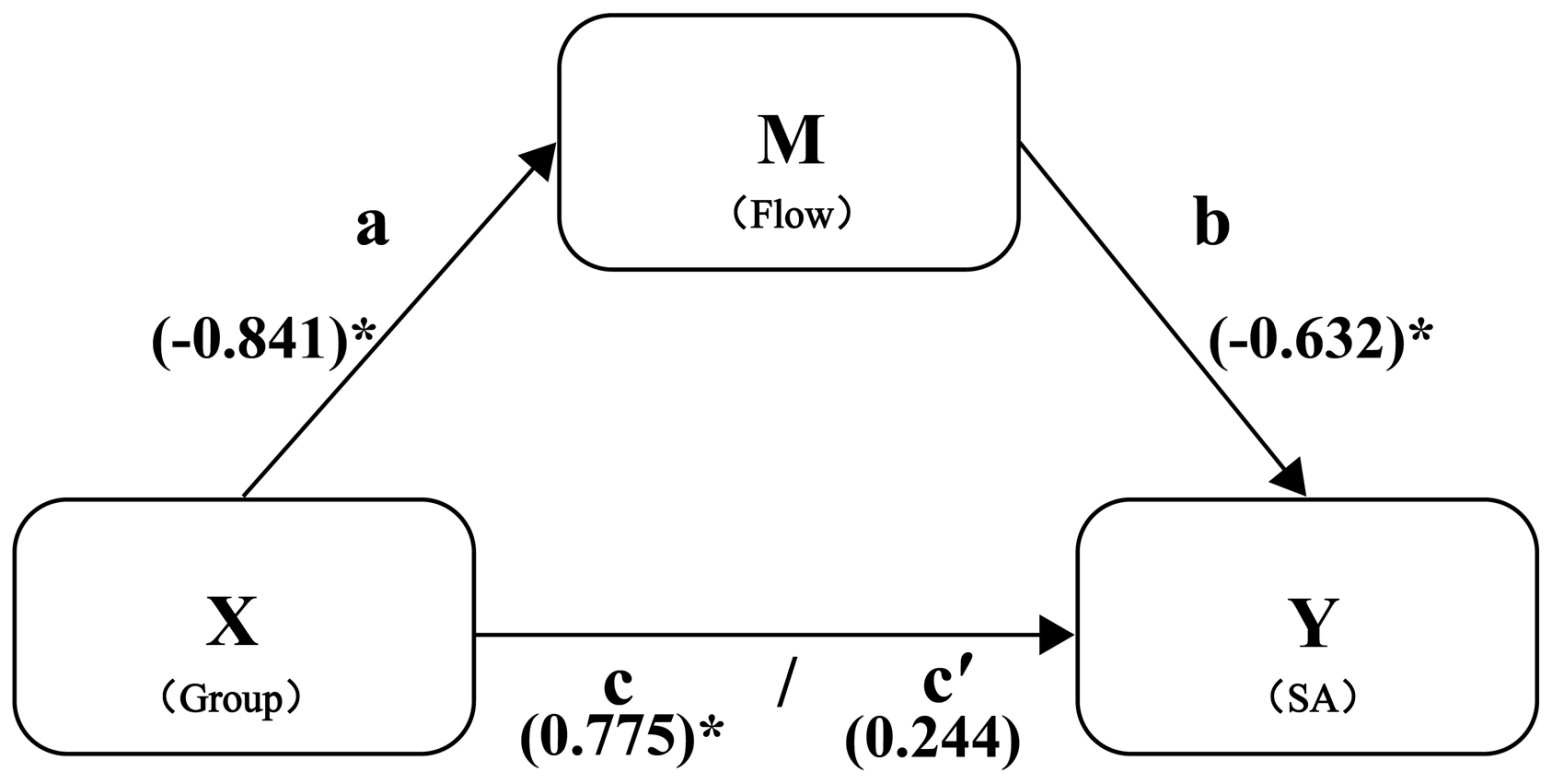
Flow state fully mediated the relationship between groups and state anxiety. The asterisk (*) denotes statistical significance, indicating that the *p*-value of the path coefficient is less than the set significance level (*p* < 0.05).

This study assessed the effect of groups of different types of mandala coloring on flow state level and state anxiety level. Results indicated that groups exerted a significant negative influence on flow state level (path a: *β* = −0.841, *p* < 0.05, CI [−1.529, −0.153]), demonstrating that group effectively predicted flow state level. Flow state level exerted a significant negative influence on state anxiety level (path b: *β* = −0.632, *p* < 0.001, CI [−0.947, −0.318]). The overall effect of group assignment on state anxiety level was also significant (path c: *β* = 0.775, *p* < 0.05, CI [0.076, 1.475]). Controlling for flow state level, the direct effect of group on state anxiety was not significant (path c′: *β* = 0.244, *p <* 0.426, CI [−0.375, 0.862]). The indirect effect of group on state anxiety level via flow state level was significant (ab = 0.532, CI [0.076, 1.250]), which suggests a full mediation. Specifically, flow state level fully mediated the relationship between group and state anxiety level (Figure 6).

## 4 Discussion

### 4.1 Anxiety therapeutic effects of digital mandala coloring

The findings of this study demonstrate that digital mandala coloring effectively reduces anxiety. This effect may be attributed to contemporary university students’ familiarity with mobile devices, and the potential of applications and such devices to elicit positive emotional responses. Previous research suggests that using electronic devices like iPad can enhance learning motivation among students (61), partly because younger generations have largely grown up in a “digital age” (62) and possess high proficiency with mobile technologies like iPad and Apple Pencil. Kinash et al. (61) further reported that most students are willing to adopt and use learning applications on iPad. Therefore, it is reasonable to conclude that mobile devices do not diminish user experience and may even offer motivational benefits for students.

Furthermore, the richer color palette and greater customization options in digital coloring may represent another contributing factor. According to *Attention Restoration Theory*, environments or activities that engage involuntary, “fascinating” attention can promote emotional recovery. Kaplan and Berman (63) identified “fascination” as a key component of such restorative environments. From the perspective of color psychology, increased color saturation and brightness can enhance viewers’ enjoyment (64). Camgöz et al. (65) experimentally confirmed that colors with the highest saturation and brightness received the highest preference ratings. Compared to physical surfaces like paper, digital mandala coloring provides colors with higher perceived saturation and adjustable brightness, which may better capture the coloring people’s attention and increase the enjoyment of the coloring process. These factors may explain why digital mandala coloring showed slightly superior anxiety reduction effects compared to the paper-based method in this study.

### 4.2 The impact of interaction mechanisms on user experience and emotional outcomes in digital mandala coloring

The results demonstrate that the sliding operation in digital mandala coloring facilitated higher level of flow experience, thereby producing more significant emotional therapeutic effects. Physiological data from participants corroborated this conclusion, showing that sliding digital mandala coloring significantly improved skin conductance levels and heart rate, with more pronounced reduction effects compared to the gamified coloring group. From a medical perspective, the sliding group exhibited clearer recovery of autonomic nervous system activity, while the gamified group required a longer duration to achieve similar relief from emotional tension.

Beyond addressing the research gap in previous digital mandala studies that relied solely on subjective measures, this study provides objective experimental evidence. The findings offer practical implications for the design and application of digital mandala coloring applications. Specifically, developers should carefully consider how different interaction modes affect user immersion, enjoyment, and psychological outcomes. This understanding can promote the integration of digital mandala coloring apps into public mental health services and advance the digital transformation of art therapy. The widespread popularity of mandala coloring books such as “Secret Garden” demonstrates substantial public interest and demand for mandala-based activities. Digital mandala applications offer equivalent therapeutic benefits while reducing material costs and procedural complexity. With lower development and distribution costs, these applications provide greater accessibility to users, presenting favorable conditions for market expansion and potential growth opportunities.

Furthermore, this research offers valuable insights for art therapy application developers focused on user experience and art therapists. This research helps correct some art therapists’ biases toward digital art interventions (66), and assists therapists in selectively employing digital mandala coloring methods during actual art therapy sessions based on clients’ specific circumstances. For future using of digital mandala coloring, careful consideration should be given to differently impact user immersion, enjoyment, and therapeutic outcomes. Such optimization could advance the integration of digital mandala coloring into public mental health services, facilitating digital transformation within art therapy. In future design and development processes, developers and designers should adequately consider the actual differences in user psychological effects generated by various interaction operations, enabling more comprehensive application optimization. In the “attention economy,” capturing users’ fragmented time represents a crucial market opportunity for application success. This study quantifies user experience as an adjustable variable, providing designers with direct pathways and strategies for enhancing user experience. This approach offers a novel perspective and carries significant practical relevance for application design and development.

### 4.3 Interpretation of physiological results

The physiological data from this study reveal a distinct autonomic response pattern elicited by digital mandala coloring. Following the coloring intervention, participants exhibited a significant decrease in skin conductance level(SCL) and heart rate (HR), indicating a reduction in sympathetic nervous system arousal, the parasympathetic nervous system plays a predominant role in regulation. At the same time, theoretically, the LF index representing sympathetic nervous activity should show a downward trend at this point, while the rMSSD index reflecting parasympathetic nervous activity should increase. This is because parasympathetic activity signifies relaxation and recovery (47), and a higher rMSSD value indicates stress relief (67). However, based on the actual experimental measurements, although the data itself showed a trend consistent with theoretical expectations, statistical analysis revealed that HRV indices (rMSSD and LF) did not show statistically significant changes. Taken together, this pattern, reduced sympathetic arousal without a marked increase in parasympathetic tone, aligns with the early-stage physiological profile observed in certain focused relaxation tasks: disengagement from a stressed state (manifested as SCL changes) may precede or occur independently from the deeper, parasympathetically dominated processes of physiological recovery.

Although the HRV data in this experiment did not reveal significant differences, this finding aligns with the characteristic that this metric is more sensitive to longer-term emotional interventions. Compared to the immediate stress sensitivity of HR and SCL, HRV reflects the regulatory function of the parasympathetic nervous system over extended periods, typically requiring more sustained interventions to observe significant changes (68). The intervention duration in this study may have been insufficient to induce structural adaptations in the autonomic nervous system.

This is not uncommon in existing research. SCL is typically suitable for experiments involving brief exposure to stressful environments and soothing materials (53). In experiments performed at the NASA-Ames Research Center, the subject’s physiological response to the stress of mental work could be concretize as a sequence of alternating high and low stress periods of SCL (54). This demonstrates that the sensitivity of SCL indicator changes aids in capturing subjects’ stress levels in an instantaneous state, further validating the immediate anxiety-relieving effects of the digital mandala.

### 4.4 The therapeutic mechanism of flow

The mediation analysis provides strong statistical evidence supporting our hypothesized mechanism. The results are consistent with a full mediation model, whereby the effect of the digital mandala coloring interactive format on reducing anxiety is statistically accounted for by increases in flow state. This offers compelling support for the theoretical role of flow as a key explanatory pathway. This indicates that digital mandala coloring alleviates anxiety by enhancing flow state, which consists with the theoretical predict proposed by Cai (35). These results validate that operational fluency, as posited by *Embodied Cognition Theory*, directly influences users’ immersion (69). They also enrich *Flow Theory* by clarifying the relationship between interactive actions in digital mandala coloring and anxiety reduction, highlighting the importance of fostering immersion in mental health applications to enhance flow experience and decrease anxiety.

Our findings support the *Embodied Cognition Theory*: The sliding interaction’s sensorimotor engagement enhances people’s flow state, which downregulates amygdala activity via prefrontal inhibition Paulus et al. (70), thereby reducing sympathetic arousal (as reflected in skin conductance level). Csikszentmihalyi et al. (27) characterize flow as a state of complete absorption in an ongoing activity, where individuals temporarily suspend “internal dialogue,” regain a sense of attentional control, and consequently disengage from negative emotions. In contrast, the animated effects, tools, and interactive features in gamified digital mandala coloring constitute frequent “task-switching” demands. Randomly occurring visual and auditory information distracts individual attention, thereby disrupting the formation of flow states.

### 4.5 Limitations and future research

This study has the following limitations. First, the sample consisted of mentally healthy college students. While this facilitates testing the core effects of digital mandala coloring while controlling for pathological interference, it limits the generalizability and applicability of the findings to clinical populations and other groups. Adolescents and older adults exhibit differences in cognitive flexibility, emotional regulation strategies, and digital native proficiency, particularly in terms of their acceptance and familiarity with digital art activities, as older adults may feel more familiar with traditional coloring (14). The user-interface of applications rarely take procedural memory, attention, and motivation of older adults into consideration. So navigating technology feels counter-intuitive to older adults (71). Low technological literacy hinders participants from achieving emotional healing effects in digital coloring activities (14). These limitations also underscore the importance of developing adaptive guidance systems. Optimizing applications to different population will allow them to take advantage of the increasing accessibility of digital self-soothing tools, including digital mandala coloring. Future research should focus on validating the robustness of these findings across different populations and exploring personalized adaptation strategies.

Although we adjusted balance of gender during subject selection, potential gender-based variations in art engagement and emotional expression should not been ignored. Investigating this factor in future research with larger, stratified samples is warranted. Besides, the reliance on self-reported anxiety measures may be subject to recall bias. And individual differences in pre-existing affinity for art or digital games could influence engagement and perceived outcomes. Future studies might incorporate objective measures like behavioral engagement metrics and assess participant predispositions to better understand their role.

Furthermore, regarding the experimental procedure, the flow state was assessed only once at a single time point (T3) following the completion of the digital mandala coloring activity. This design decision was based on the consideration that flow is not an instantaneous state but typically requires a period of sustained immersion to become fully established. Therefore, a post-intervention assessment was deemed more appropriate for capturing the overall flow experience elicited by the activity, as interrupting the coloring process to measure flow mid-task could disrupt the natural development of the state and introduce measurement artifacts. Additionally, the primary focus of this study was on comparative analysis between groups, for which a single, well-timed measurement facilitates clearer statistical comparisons.

However, it is important to acknowledge that while this single-timepoint measurement effectively captured the aggregate flow experience and its established association with anxiety reduction, it inherently limited the exploration of dynamic changes in flow during the intervention. Specifically, this design cannot elucidate when the flow state commenced, when it peaked, or how its temporal trajectory correlated with the real-time alleviation of anxiety. Future research would benefit from employing more granular assessment strategies. These could include embedding multiple brief assessments using the *Experience Sampling Method* during the activity or incorporating continuous physiological measures (e.g., EEG, SCL) to map the dynamics of the flow state with greater precision and to further disentangle its momentary effects as a mediating mechanism.

In addition, from a mechanistic perspective, the current results suggested that coloring interventions may primarily exert immediate effects through activation of the sympathetic nervous system, with limited impact on parasympathetic pathways. This also highlights a key limitation of the present study. Future investigations should implement periodic long-term interventions to further validate the psychological therapeutic effects of digital mandala coloring.

## 5 Conclusion

Through psychophysiological experiments, this study systematically investigated the intervention effects of digital mandala coloring on anxiety among university students and its underlying mechanisms. The results supported the following conclusions:

1. Digital mandala coloring effectively alleviates state anxiety in university students and reduces their heart rate and skin conductance level, with no significant difference to the traditional method.
2. Digital mandala coloring exhibits advantages in physiological measures. It shows significantly stronger and more efficient calming effects on users’ heart rate compared to the paper-based method, alongside eliciting a higher level of flow state.
3. The interaction mode used in digital mandala coloring influences the therapeutic experience and emotional outcomes. Overall, sliding coloring demonstrates significantly superior anxiety reduction and flow state compared to gamified coloring. This conclusion is further supported by physiological data, with sliding coloring producing significantly greater reductions in both heart rate and skin conductance level.
4. These results are attributed to the positive effects of flow state. Flow state mediates the relationship between digital mandala coloring activities and anxiety reduction, completely influencing state anxiety level.

This research provides valuable insights for both art therapy and the development of mandala coloring research. It concretely applies flow theory to the field of digital art therapy, deepening our understanding of its mechanism of action. Unlike previous studies on traditional mandala coloring for anxiety relief, this research identifies and examines key factors (e.g., interaction mode) that affect anxiety reduction in the digital context as adjustable design variables. By elucidating the dynamic process of user experience and its underlying mechanisms, the findings offer designers direct pathways and actionable strategies for user experience improvement and providing art therapists a novel perspective with significant practical implications.

On a practical level, mental health interventions for university students can effectively leverage the advantages of mobile digital media and products. Artistic interaction activities characterized by high operational fluency and high concentration will serve as effective intervention measures. The findings provide direct and crucial optimization directions for the design of art therapy activities, particularly mandala coloring apps. Art therapy activities should prioritize interaction that enhances operational fluency and immersion rather than solely pursuing entertainment value and complexity, thereby maximizing their emotional therapeutic potential.

## Data Availability

The datasets presented in this article are not readily available because the data analyzed in this study is subject to the following licenses/restrictions: the data is supporting other study which is unpublished. The data supporting the findings of this study can be obtained upon request from the corresponding author. However, the data are not publicly accessible due to privacy and ethical considerations. Requests to access these datasets should be directed to ynli19@163.com.
